# Health-Promoting Effects of Black Tea: A Narrative Review of Clinical Trials

**DOI:** 10.1155/ijfo/8560718

**Published:** 2025-02-18

**Authors:** Yusuf Yilmaz

**Affiliations:** Department of Gastroenterology, School of Medicine, Recep Tayyip Erdoğan University, Rize, Türkiye

**Keywords:** antioxidant, black tea, cardiovascular health, clinical trials, inflammation, metabolic health, microbiota

## Abstract

Black tea, a popular beverage, is rich in polyphenols. However, thorough analyses of clinical trial evidence supporting its health benefits are lacking. This narrative review is aimed at addressing this knowledge gap by synthesizing findings across pivotal clinical domains and identifying critical areas for further investigation. A comprehensive search of PubMed/MEDLINE, PubMed Central, Scopus, Web of Science, and Google Scholar was performed for English-language papers from January 1990 to July 2024, focusing on cardiovascular and metabolic health, as well as cognitive function. Findings from clinical trials indicated that consuming black tea regularly enhances endothelial and vascular health, notably by improving flow-mediated vasodilation. These advantages are largely due to the tea's antioxidant, anti-inflammatory, and gut microbiota–modulating effects, including the promotion of beneficial bacterial species such as *Flavonifractor plautii*. Effects on metabolic health, such as lipid profiles and glucose metabolism, were inconsistent. However, black tea was linked to improved cognitive function, especially attention and alertness, likely due to caffeine and L-theanine. Despite these promising results, further research is needed to overcome limitations like small sample sizes and short study durations. Future studies should be aimed at standardizing black tea preparations to optimize health benefits.

## 1. Introduction

Tea, which is obtained from the young shoots or leaves of the *Camellia sinensis* plant, is the second most widely consumed beverage globally, after water, and is appreciated for its unique sensory qualities and potential health benefits [1, 2]. Among the diverse varieties available, black tea, characterized by complete fermentation and oxidation during processing, dominates the market with an approximately 78% share [3, 4]. This prevalence, coupled with emerging evidence of its health-promoting effects, has positioned black tea as a key focus in nutritional epidemiology and clinical investigations.

The potential health advantages of black tea are predominantly linked to its abundant polyphenol composition, specifically flavonoids, including catechins, theaflavins, and thearubigins [5, 6]. These bioactive constituents, along with caffeine, methylxanthines, vitamins, and volatile compounds, may exert antioxidant, anti-inflammatory, and microbiota-modulating effects [7]. Notably, epidemiological evidence, particularly from large-scale longitudinal studies, has provided support for tea's positive health effects, specifically in terms of mortality reduction. In this regard, a prospective cohort study utilizing data from the UK Biobank, encompassing 498,043 participants (age range: 40–69 years) over a median follow-up of 11.2 years, revealed a significant inverse association between higher tea intake and mortality risk [8]. Specifically, the consumption of two or more cups daily was significantly associated with lower mortality rates, independent of genetic variations in caffeine metabolism [8]. Subsequent analyses of the same dataset have yielded additional insights. Chen et al. [9] reported a reverse J-shaped association with all-cause mortality, with the nadir of risk observed at three cups per day. In a separate investigation, Wu et al. [10] revealed a notable inverse correlation between tea consumption and all-cause and cancer mortality among patients with metabolic syndrome, with a daily intake of four or more cups linked to an 11% decrease in cardiovascular mortality.

While these epidemiological findings are noteworthy, clinical trials remain the gold standard for evaluating interventions in evidence-based medicine [11]. Despite numerous authors exploring potential health benefits of tea consumption in humans [12–15], there is a notable lack of comprehensive analyses examining clinical trial evidence specifically pertaining to black tea. This narrative review is aimed at addressing this knowledge gap by synthesizing findings across pivotal clinical domains and identifying critical areas for further investigation.

## 2. Materials and Methods

**Figure 1:**
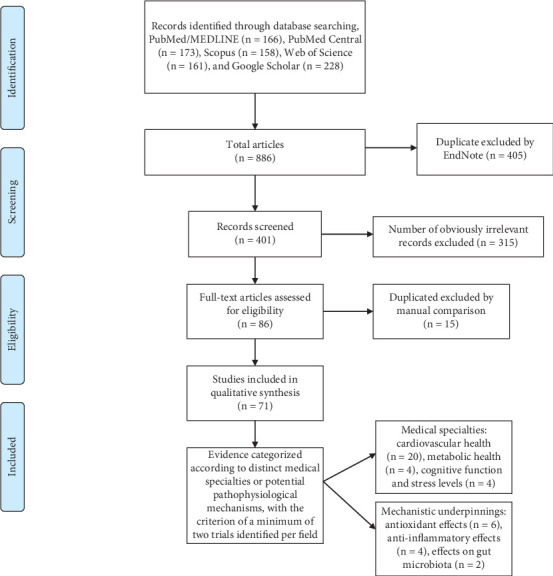
Flow diagram of studies' screening and selection.

A comprehensive review was performed across multiple academic databases, including PubMed/MEDLINE, PubMed Central, Scopus, Web of Science, and Google Scholar, focusing on English-language clinical trials published from January 1, 1990, to July 1, 2024. The search strategy employed the following terms: “black tea” AND (“clinical trial” OR “randomized controlled trial”) (Table 1). The search yielded 86 articles related to clinical trials or randomized controlled trials that investigated the effects of black tea consumption on human health. Full-text articles were reviewed, and data synthesis was conducted through a two-phase narrative approach. Initially, evidence was aggregated and categorized according to distinct medical specialties, with the criterion of a minimum of two trials identified per field. Through collaborative consensus among the investigators, three primary categories emerged from the analyzed literature: (1) cardiovascular health, (2) metabolic health, and (3) cognitive function/stress levels. Subsequently, data were extracted from trials investigating potential pathophysiological mechanisms underlying the putative health benefits of assuming black tea. This encompassed examinations of its effects on antioxidant status, inflammatory biomarkers, and gut microbiota composition. A flow diagram of studies' screening and selection is presented in Figure 1.

## 3. Results

### 3.1. Clinical Trials Assessing Black Tea in Relation to Cardiovascular Health

In the realm of cardiovascular health research, black tea has emerged as a subject of significant interest, with clinical trials focusing on its effects across three critical domains, that is, (1) endothelial and vascular function, (2) cholesterol and lipid profile, and (3) platelet aggregation and hemostasis (Table 2).

#### 3.1.1. Endothelial and Vascular Function

Endothelial dysfunction plays a pivotal role in the development and advancement of atherosclerosis, potentially contributing to the onset of hypertension and cardiovascular complications [31]. Several clinical trials have examined the potential of black tea intake to enhance endothelial function in both healthy individuals and clinical populations. In a landmark investigation, Duffy et al. [16] performed a randomized crossover trial encompassing 66 patients with coronary artery disease to evaluate the acute and chronic effects of black tea consumption on endothelial function. They found that both acute (2 h after 450 mL) and chronic (900 mL daily for 4 weeks) black tea consumption significantly improved flow-mediated dilation (FMD) of the brachial artery, indicating enhanced endothelium-dependent vasodilation. This improvement was observed in contrast to water consumption, which had no effect, and was not attributable to caffeine alone, as a separate group of patients receiving a 200-mg caffeine dose—equivalent to the caffeine content in 450 mL of brewed tea—showed no significant FMD modifications. Additionally, the improvement in endothelial function correlated with increased plasma catechin levels after tea consumption, suggesting that tea flavonoids, rather than caffeine, were responsible for the observed vascular benefits [16]. Hodgson et al. [17] corroborated and expanded upon the findings of Duffy et al. [16] in a study involving 21 subjects with mildly elevated cholesterol levels. The authors investigated the impact of regular black tea consumption, specifically five cups (250 mL) per day for 4 weeks, on brachial artery function. Their results showed an increase in endothelium-dependent dilatation by 2.3%, supporting prior observations [17]. Notably, the authors also observed a 4.2% enhancement of endothelium-independent vasodilation, suggesting that black tea components may have direct effects on vascular smooth muscle in addition to endothelial cells [17]. These findings are particularly important as they provide evidence for multiple mechanisms of action by which black tea may promote vasodilation. In their double-blind, controlled, crossover randomized trial conducted on healthy males, Grassi et al. [18] demonstrated that FMD increased and peripheral arterial stiffness decreased in a dose-dependent fashion after black tea consumption. Importantly, the effects were observed even doses as low as 100 mg/day (i.e., less than one teacup), suggesting that even moderate tea consumption may confer benefits in terms of vascular function. This research provided valuable insights into the potential threshold for cardiovascular benefits from black tea consumption. Importantly, the authors also observed that black tea consumption significantly decreased both systolic (−2.6 mmHg) and diastolic (−2.2 mmHg) blood pressure. These findings were subsequently expanded by Hodgson et al. [19], who conducted a longer-term study investigating the effects of regular black tea consumption on blood pressure variability over 6 months. In a randomized, double-blind, placebo-controlled trial with 111 subjects having systolic blood pressure between 115 and 150 mmHg, the authors reported that consuming three cups of powdered black tea daily led to reduced systolic and diastolic blood pressure variability during nighttime. The observed effects were both immediate upon initial consumption and consistent throughout a 6-month period, occurring independently of baseline blood pressure and heart rate levels [19]. The study is notable for its longer duration, which provides insights into the sustained effects of tea consumption. Additionally, the focus on blood pressure variability is significant, as this parameter has been associated with cardiovascular risk independently of average blood pressure levels [32]. Interestingly, in a separate randomized controlled trial conducted by the same authors [33], it was observed that regular consumption of black tea over a period of 6 months resulted in a reduction in blood pressure. However, the acute 24-h ambulatory blood pressure measurements remained unchanged, indicating that the beneficial long-term effects of black tea consumption are likely mediated by mechanisms that are independent of immediate blood pressure regulation. This suggests that the observed vascular benefits may necessitate sustained exposure to tea polyphenols, allowing for the cumulative effects of bioactive metabolites to modify vascular function over time. Schreuder et al. [20] investigated the acute effects of black tea on endothelial function in 20 healthy subjects, with a particular focus on its potential protective effects against ischemia–reperfusion (IR) injury. Their study demonstrated improved FMD following a week of black tea consumption (three cups per day), consistent with previous findings [16–18]. However, black tea did not protect against IR-induced endothelial dysfunction [20]. This research is relevant as it highlights the complexity of black tea's cardiovascular effects and suggests that while tea may improve baseline endothelial function, its protective effects against IR may have limitations. In a randomized, double-blind, controlled, crossover study, Grassi et al. [21] investigated the effects of black tea on circulating angiogenic cells (CACs) and FMD in 19 hypertensive patients. The results revealed that black tea consumption significantly increased the number of functionally active CACs and improved FMD compared to a placebo. Black tea also counteracted the decrease in FMD caused by a fat challenge, demonstrating its vascular protective properties [21]. This research is particularly significant as it demonstrates potential benefits in a population at higher cardiovascular risk. The study also provides insights into the acute vascular effects of tea consumption, suggesting that tea might be beneficial when consumed with meals. However, Greyling et al. [22] conducted a study that yielded results contrasting with some previous findings. This randomized controlled trial investigated the acute effect of black tea consumption on forearm resistance artery endothelial function in healthy, middle-aged subjects. Twenty participants consumed either black tea containing approximately 400 mg of flavonoids or a placebo, and their forearm blood flow responses to various substances were measured 2 h later. Although the mean forearm blood flow response to acetylcholine was 23% higher after tea consumption compared to placebo (95% confidence interval (CI): −20%, +88%), this difference did not achieve statistical significance (*p* = 0.32). While this magnitude of change could suggest potential physiological relevance, the wide CIs and high variability in responses warrant careful interpretation of its clinical significance. The authors concluded that acute intake of black tea did not significantly alter endothelium-dependent vasodilation of forearm resistance arteries in healthy middle-aged subjects [22]. This study notably highlights the variability in research outcomes and the complexity of black tea's cardiovascular effects. It also suggests that the benefits of tea consumption may depend on various factors, including the specific vascular bed studied, the population characteristics, and the dosage and duration of tea consumption. In a separate trial, Woodward et al. [23] expanded the focus of black tea research cutaneous microvascular function in healthy middle-aged adults. Specifically, 20 participants underwent two experimental trials, consuming either 200 mL of black tea or a placebo, followed by assessment of forearm cutaneous perfusion using laser-Doppler flowmetry and full-field laser perfusion imaging. The results showed that gradual local heating to 42°C produced higher skin blood flow following black tea ingestion, as measured by absolute cutaneous vascular conductance and flux. The researchers concluded that acute tea ingestion enhanced cutaneous vascular responses to gradual local heating, possibly through activation of endothelium-derived chemical mediators like nitric oxide, which may contribute to the cardiovascular health benefits of regular tea consumption [23]. Intriguingly, these findings suggest potential benefits of tea consumption for microvascular function, an important aspect of cardiovascular health that had received less attention in previous studies. In a randomized, controlled, crossover study with 17 healthy volunteers over 4-week periods, Ahmad et al. [24] have recently examined the impact of black tea, with and without milk, on vascular function and blood pressure. Black tea alone was found to enhance FMD compared to hot water, whereas black tea with milk reduced FMD and elevated blood pressure. Both tea preparations lowered heart rate compared to hot water, but plasma nitrate and nitrite levels remained unchanged across treatments [24]. These findings highlight the potential modulation of tea's cardiovascular benefits when consumed concomitantly with milk, underscoring the importance of considering the interaction effects as a potential confounding variable in future studies.

#### 3.1.2. Cholesterol and Lipid Profile

Dyslipidemia plays a pivotal role in the pathophysiology of cardiovascular disease, necessitating the identification of nonpharmacological interventions as complementary therapeutic strategies [34, 35]. In this context, the potential efficacy of black tea consumption has been the subject of clinical investigation. In a randomized, controlled crossover study including 15 mildly hypercholesterolemic adults (mean baseline total cholesterol: 208.9 ± 5.4 mg/dL) conducted by Davies et al. [25], participants consumed five servings of black tea daily for 3 weeks, compared to a caffeine-matched placebo. The study demonstrated significant reductions in several lipid parameters with black tea consumption. Specifically, total cholesterol decreased by 6.5%, LDL cholesterol by 11.1%, apolipoprotein B by 5%, and lipoprotein(a) by 16.4%. However, no significant changes were observed in HDL cholesterol, apolipoprotein A-I, or triglyceride levels [25]. In line with these findings, Fujita and Yamagami [26] found that the ingestion of black tea extract (BTE) tablets significantly reduced total cholesterol, LDL cholesterol, and triacylglycerol levels in subjects with borderline hypercholesterolemia (baseline total and LDL cholesterol levels ranging from 4.65 to 6.72 mmol/L and 2.59 to 4.14 mmol/L, respectively) after 3 months. In 2012, Bahorun et al. [27] conducted a 12-week randomized controlled trial that included 87 subjects who consumed three cups of black tea daily. The results demonstrated significant decreases in LDL/HDL cholesterol ratio (16.6%) and triglyceride levels (35.8%), along with a nonsignificant increase in HDL cholesterol (20.3%) [27]. While these findings suggest potential benefits of black tea consumption on lipid profiles, the results have not been invariably consistent. In their 6-month randomized trial involving 31 older adults with cardiovascular risk factors who consumed three cups of black tea daily or a control beverage, Mukamal et al. [36] found no significant impact on lipid profiles in the tea group compared to the control group. In a separate study, Trautwein et al. [37] investigated the effects of specific black tea components on cholesterol levels in 102 mildly hypercholesterolemic subjects (baseline total cholesterol and LDL cholesterol: 5.70 ± 0.74 and 3.97 ± 0.61 mmol/L, respectively). Participants consumed either purified black tea theaflavins or a theaflavin/catechin combination daily for 11 weeks. The authors compared these interventions to a placebo group. However, no significant effects on serum lipids were observed for specific tea components compared to placebo [37]. In another randomized crossover trial that involved 57 subjects with borderline hypercholesterolemia (baseline total cholesterol concentrations: 190–260 mg/dL), participants consumed five cups of black tea daily for 4 weeks, compared to a placebo, while following a strictly controlled low-flavonoid diet [38]. The controlled diet consisted of 51% carbohydrate, 15% protein, and 34% fat, with no fruits and minimal vegetables to eliminate dietary flavonoid interference. Despite the rigorous dietary control and relatively high tea intake, no significant effects on total, LDL, or HDL cholesterol levels compared to placebo were observed [38]. These findings contribute to the existing body of conflicting evidence concerning the effects of black tea consumption on serum lipid profiles. The inconsistencies observed across studies may be attributed to several factors, including variations in experimental design, intervention duration, tea dosage, and participant demographics. Such heterogeneity in research parameters underscores the complexity of elucidating the precise relationship between black tea intake and lipid metabolism.

#### 3.1.3. Effects on Platelet Aggregation and Hemostasis

In light of the pivotal role platelets and hemostasis-related markers play in the initiation of atherothrombotic events [39], researchers have conducted trials to examine the potential antiplatelet effects of black tea. In their randomized crossover study, Hodgson et al. [28] investigated the effects of regular black tea consumption on hemostasis-related variables and cell adhesion molecules. Twenty-two subjects consumed either five cups of black tea or hot water daily for 4-week periods. The results revealed lower soluble P-selectin levels with black tea consumption compared to hot water, suggesting a potential antiplatelet effect. However, no significant differences were observed in platelet aggregation responses to collagen or ADP between the tea and water interventions [28]. A subsequent study by the same research group examined the acute effects of black tea on postprandial platelet aggregation in 20 healthy subjects [29]. Participants consumed 50 g of dairy fat followed by either black tea or hot water. The findings showed no significant differences in collagen- or ADP-induced platelet aggregation between the black tea and hot water treatments 4 h postconsumption [29]. In another 6-week study on 75 healthy men, Steptoe et al. [30] compared the effects of black tea to placebo on platelet activation. The results revealed no significant differences in platelet activation markers between the tea and placebo groups. Collectively, these findings suggest that while black tea consumption may influence certain hemostasis-related markers, such as soluble P-selectin, its effects on platelet aggregation and activation are likely negligible.

### 3.2. Clinical Trials Assessing Black Tea in Relation to Metabolic Health

In the field of metabolic health, clinical trials on black tea have focused on two main domains, that is, (1) glucose metabolism/diabetes mellitus and (2) body weight (Table 3).

#### 3.2.1. Effects on Glucose Metabolism and Diabetes Mellitus

In 2007, Bryans, Judd, and Ellis [40] conducted a randomized, crossover trial involving 16 healthy adults to assess the effects of black tea consumption on postprandial glucose and insulin responses. Participants consumed either 250 mL of water (control), 250 mL of water plus 0.052 g of caffeine (positive control), or 250 mL of water plus 1.0 g of instant black tea. The results revealed that plasma glucose concentrations were significantly reduced at 120 min following ingestion of the 1.0-g tea drink compared to the control and caffeine drinks. In addition, black tea consumption resulted in increased insulin concentrations compared to the control and caffeine drinks at 90 min and compared to the caffeine drink alone at 150 min [40]. In a randomized, double-blind, placebo-controlled crossover study involving 24 subjects with normal and prediabetic glucose levels, participants randomly ingested a sucrose solution with a low dose (110 mg black tea polymerized polyphenol (BTPP)) and a high dose (220 mg BTPP) of black tea drink or a placebo drink (0 mg BTPP) [41]. The study revealed that BTPP significantly reduced incremental blood glucose area under the curve following sucrose consumption in both normal and prediabetic participants. While insulin levels showed no statistically significant variations between the placebo and black tea groups, no notable adverse effects were detected across different BTPP dosages. The researchers concluded that black tea intake can effectively lower postprandial blood glucose levels after sucrose intake [41]. A separate 3-month randomized controlled trial to evaluate the impact of green and BTE supplementation on 49 adults with Type 2 diabetes [42]. Participants were randomly assigned to consume either 0, 375, or 750 mg/day of the tea extract for 3 months. The primary endpoint was change in glycated hemoglobin at 3 months. After the intervention period, the mean changes in glycated hemoglobin were +0.4%, +0.3%, and +0.5% in the placebo, 375-mg, and 750-mg arms, respectively. However, these changes were not significantly different between study arms. The authors concluded against a hypoglycemic effect of extract of green and black tea in adults with Type 2 diabetes mellitus [42].

#### 3.2.2. Effects on Body Weight

Obesity and overweight are widely recognized as critical public health challenges in modern medicine [44]. Given their prevalence and associated health risks, researchers have directed significant attention towards investigating potential complementary and alternative medicine interventions, with black tea emerging as a subject of interest in clinical trials. However, published results have been inconsistent. In their 12-week randomized controlled trial involving 87 subjects, Bahorun et al. [27] demonstrated that daily consumption of three cups of black tea did not lead to significant changes in body weight or BMI compared to the control group. Similarly, Troup et al. [38] reported that consumption of five cups/day of black tea for 4 weeks did not yield significant effects on body weight or BMI compared to placebo in a study involving 57 individuals with borderline hypercholesterolemia. Conversely, Bøhn et al. [43] reported different findings in a randomized, controlled, double-blind 6-month parallel study with 111 healthy adults. Participants consumed either three cups/day of black tea or a caffeine-matched placebo. Compared to placebo, black tea intake inhibited weight gain (−0.64 kg, *p* = 0.047), reduced waist circumference (−1.88 cm, *p* = 0.035), and decreased waist-to-hip ratio (−0.03, *p* = 0.005) over 3 months. However, these effects were no longer significant at the 6-month mark [43].

### 3.3. Clinical Trials Assessing Black Tea in Relation to Cognitive Function and Stress Levels

Four clinical trials have explored the effects of tea consumption on cognitive function, mood, and stress levels (Table 4). Hindmarch et al. [45] found that black tea improved accuracy on attention tasks and increased self-rated alertness compared to placebo (no caffeine). In addition, tea drinking throughout the day significantly the diurnal pattern of performance decrements found with the placebo condition [45]. Building on these findings, De Bruin et al. [46] conducted a double-blind, randomized, placebo-controlled crossover trial that demonstrated black tea's ability to enhance accuracy on switching attention tasks and boost self-reported alertness. Okello, Abadi, and Abadi [47] expanded the scope of research by investigating the effects of both green and black tea on brain wave activities using EEG on eight healthy volunteers. Their results revealed increased alpha, theta, and beta wave activities 1 h after tea consumption, with green tea showing a particularly significant increase in theta waves. This study highlighted the potential neurophysiological effects of tea consumption, although considerable interindividual variability was observed [47]. Focusing on stress reduction, Yoto et al. [48] explored the effects of black tea aroma. Their study showed that inhaling black tea aroma inhibited the increase in salivary chromogranin-A, a stress marker, following arithmetic stress tasks. Additionally, black tea aroma tended to decrease tension and anxiety scores immediately after exposure [48]. Collectively, these studies suggest that tea consumption may offer beneficial effects on cognitive function, alertness, and stress reduction.

### 3.4. Mechanistic Underpinnings of Black Tea's Health Effects

The mechanistic underpinnings of black tea's health effects have been examined in several human clinical trials, focusing on its impact on antioxidant status, inflammatory markers, and gut microbiota (Table 5).

#### 3.4.1. Antioxidant Effects

While black tea has the potential to exert antioxidant effects, this activity has been shown to be influenced by several factors. In a seminal study, Langley-Evans [49] demonstrated that consuming black tea without milk led to a substantial increase in plasma antioxidant potential, measured at 65%–76% over a 6-h period in healthy subjects. Interestingly, when milk was added to the tea, no significant change in antioxidant potential was observed [49]. This observation aligns with the findings by Ahmad et al. [24], who reported similar effects on vascular function and blood pressure, suggesting that milk may attenuate the positive impacts of black tea. However, other studies have yielded mixed results regarding the effects of black tea on oxidative stress markers. O'Reilly et al. [56] found no significant effects of a high-flavonoid diet, including black tea, on plasma F2-isoprostanes—a marker of lipid peroxidation—or antibodies to oxidized LDL compared to a low-flavonoid diet in healthy subjects. Widlansky et al. [51] observed that both acute and chronic black tea consumption increased plasma catechin levels but did not affect systemic markers of oxidative stress in 66 patients with coronary artery disease. In contrast, Bahorun et al. [27] demonstrated that regular consumption of black tea for 12 weeks led to a substantial 418% increase in plasma antioxidant capacity in healthy subjects. Finally, a randomized controlled trial on 46 patients with Type 2 diabetes investigated the effects of varying doses of BTE over a 4-week period [50]. The test group received increasing doses of BTE (150, 300, 450, and 600 mL) during Weeks 1–4, while the control group consistently received 150-mL BTE daily. Both groups showed similar increases in serum total antioxidant capacity. Consuming 300-mL BTE daily exerted a suppressing effect on serum malondialdehyde levels; in addition, 600-mL BTE daily significantly increased glutathione levels [50].

#### 3.4.2. Anti-Inflammatory Effects

Akin to its antioxidant properties, clinical trials have explored black tea's impact on inflammatory and immune markers. However, findings remain inconclusive due to inconsistent results across studies. On the one hand, Bahorun et al. [52] conducted a 12-week randomized controlled trial involving 30 patients diagnosed with Type 2 diabetes to investigate the effects of black tea consumption on C-reactive protein (CRP) levels. The intervention group consumed 9 g of black tea daily (equivalent to three cups) without additives, whereas the control group consumed an equal volume of hot water. In the black tea group, CRP levels in high-risk patients (> 3 mg/L) were found to decrease significantly, with a 53.4% reduction in men and a 41.1% reduction in women [52]. In patients with Type 2 diabetes, black tea consumption was associated with increased regulatory T cells (CD3+ CD4+ CD25+ FOXP3) and immunosuppressive CD3+ CD4+ IL-10+ cells, alongside reduced proinflammatory CD3+ CD4+ IL-17+ cells and Th1-associated CD3+ CD4+ IFN-*γ*+ cells [57]. These findings, coupled with those of Neyestani et al. [50] on CRP levels, suggest potential anti-inflammatory and immunomodulatory effects of black tea in Type 2 diabetes patients. On the other hand, Widlansky et al. [51] found no effect of acute (2 h) or chronic (4 weeks) black tea consumption on CRP concentrations in patients with coronary artery disease. Similarly, de Maat al. [53] observed no significant changes on inflammatory markers after 4-week administration of black tea, green tea, green tea polyphenol isolate, and mineral water in 64 apparently healthy smokers. These discrepancies in findings may be attributed to differences in study populations, duration, and design.

#### 3.4.3. Effects on Gut Microbiota

Black tea's potential to exert positive health effects through gut microbiota metabolism and modulation stands out as one of its most intriguing properties. van Duynhoven et al. [54] conducted a pilot randomized, placebo-controlled crossover trial employing an untargeted liquid chromatography tandem mass spectrometry–based metabolomics approach. This study, involving 12 healthy men, revealed a kinetic response for 59 black tea polyphenol metabolites in plasma. The findings demonstrated rapid absorption of conjugated and unconjugated catechins within 2–4 h, followed by the appearance of microbial catabolites. These results suggest that the swift and sustained circulation of conjugated catabolites produced by gut microbiota may be particularly relevant to the proposed health benefits of black tea [54]. Based on these findings, it is feasible that individual differences in gut microbiota catabolic capacity may influence the presence and effects of these metabolites. Another recent trial has illuminated the complex interplay between black tea consumption and gut microbiota composition, extending beyond the role of gut microbiota in catechin metabolism [55]. In this regard, a randomized, single-blind, 12-week placebo-controlled study conducted by Tomioka et al. [55], involving 72 healthy Japanese adults, has revealed significant modifications in the gut microbiota, characterized by an increased abundance of *Prevotell*a bacteria and a concomitant decrease in fecal acetic acid concentration. Furthermore, in participants with low salivary secretory immunoglobulin A (sIgA) levels, black tea intake was associated with an increase in total bacteria, elevated *Prevotella* abundance, and higher levels of butyrate-producing bacteria [55]. Of particular interest was the observation that in subjects with a low abundance of the butyrate-producing *Flavonifractor plautii*, black tea consumption led to a significant increase in both salivary sIgA concentration and the absolute number of *F. plautii* [55]. These findings underscore the intricate relationship between black tea polyphenols and gut microbiota, presenting a fascinating avenue for further research with potential implications for human health.

## 4. Discussion

**Figure 2:**
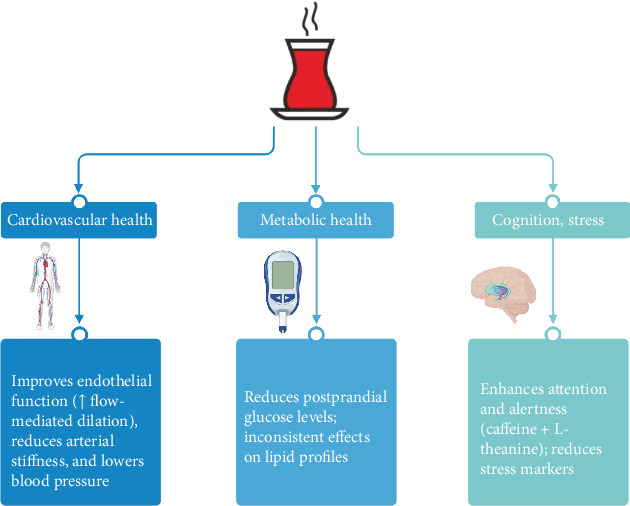
Key effects of black tea on cardiovascular health, metabolic health, cognition, and stress. Created with Biorender.com.

This narrative review of clinical trials and randomized controlled studies examining the effects of black tea on human health demonstrates its potential to confer significant benefits, particularly in the domains of cardiovascular and metabolic health, cognitive function, and stress modulation (Figure 2).

**Figure 3:**
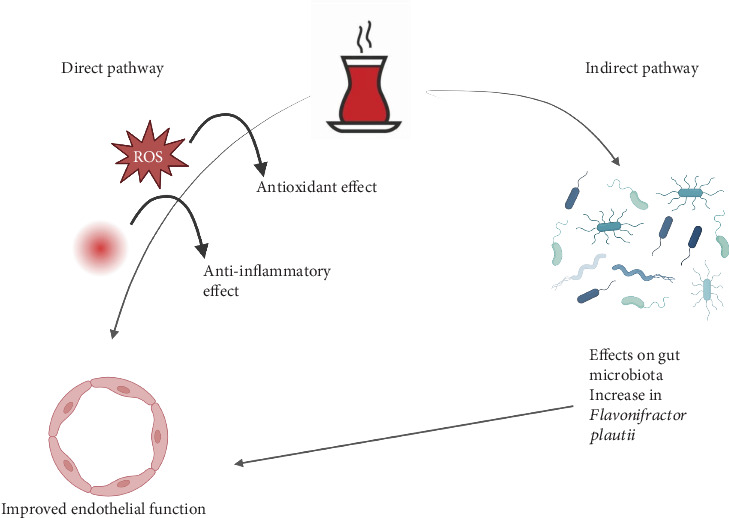
Potential pathways through which black tea consumption improves endothelial function. The direct pathway highlights the anti-inflammatory and antioxidant effects of tea polyphenols, which improve vascular health by reducing oxidative stress and inflammation. The indirect pathway emphasizes black tea's impact on gut microbiota, particularly the increase in *F. plautii*, a bacterium associated with reduced arterial stiffness. Created with Biorender.com.

Among the most reproducible findings across multiple investigations is black tea's positive influence on blood vessel health and endothelial function [16–21, 31–33]. Accordingly, clinical trial evidence supporting both acute and chronic black tea consumption's ability to significantly improve FMD can be considered robust [16–18, 20, 21]. Notably, these effects appear to be dose-dependent and have been observed even at moderate levels of tea intake [18]. While the vascular benefits have been traditionally attributed to the antioxidant and anti-inflammatory effects of tea polyphenols on endothelial cells and vascular smooth muscle, we propose that these benefits may also result from black tea's microbiota-modulating effects (Figure 3).

**Figure 4:**
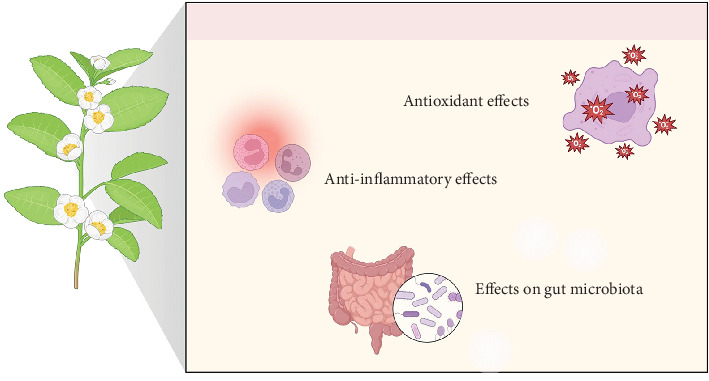
Multifaceted mechanisms of black tea's action. Black tea consumption has been shown to enhance plasma antioxidant potential, although this effect can vary based on preparation methods and study designs. Notably, the addition of milk may reduce these benefits. Clinical trials also suggest that black tea may reduce biomarkers of inflammation, including CRP, and modulate immune cell populations, potentially offering benefits for individuals with conditions like Type 2 diabetes. Finally, black tea polyphenols interact with gut microbiota, influencing the activity and composition of microbial communities. *Created with Biorender.com*.

Specifically, black tea may increase the relative abundance of *F. plautii* [55], a species known to reduce arterial stiffness by suppressing matrix metalloproteinase-2 and inhibiting nuclear factor kappa-B activation and monocyte chemoattractant protein-1 [58]. This may also explain the significant decrease in blood pressure values [18] as well as blood pressure variability [19] observed following black tea intake. Regarding lipid profiles, the effects of black tea have been more variable across studies. While some trials reported significant reductions in triglyceride, LDL cholesterol, and total cholesterol with tea consumption [25–27], others found no significant changes [36–38]. These discrepancies are likely the results of differences in study populations, tea dosages, and trial durations. Nevertheless, the potential lipid-lowering effects of black tea warrant further investigation, particularly in individuals with dyslipidemia. Conversely, the reviewed evidence suggests that platelet aggregation and hemostasis play a minor role in black tea's cardiovascular health-promoting effects [28–30]. In terms of glucose metabolism, the clinical trial data appear controversial. While black tea consumption can significantly reduce postprandial glucose levels and improve insulin sensitivity, this effect appears limited to individuals who are either nondiabetic or prediabetic [40, 41]. Accordingly, a 3-month trial in individuals with diabetes did not identify significant reductions in glycated hemoglobin levels [42], indicating that the evidence in this area remains inconclusive. Similarly, regarding body weight, the current trial evidence suggests that black tea's effect on weight reduction is likely negligible [27, 38, 43]. Conversely, another important finding from our review is the evidence that black tea can significantly enhance accuracy on attention tasks and increase self-reported alertness [45–47]. The cognitive enhancement [45–47] and stress-reducing [48] properties observed with tea consumption are mainly linked to its key bioactive ingredients, notably caffeine and L-theanine [59]. While caffeine is recognized for its ability to enhance alertness, L-theanine offers complementary effects by fostering relaxation and alleviating stress [59]. The differing concentrations of caffeine and L-theanine in various black tea preparations may account for the beverage's ability to produce these seemingly opposing effects. In addition to the well-documented impacts of these two molecules on cognitive function and stress, the clinical trial results underscored the complex and not fully understood mechanisms driving the health benefits of black tea, especially its antioxidant, anti-inflammatory, and gut microbiota–modulating effects (Figure 4).

Research on black tea's influence on antioxidant status revealed a nuanced picture. Several trials have demonstrated significant increases in serum or plasma antioxidant potential [27, 49, 50]. However, these effects seem to be partially dependent on factors such as the presence of additives like milk [49]. Conversely, other studies have reported negative findings [51, 56]. The question of whether these discrepancies are at least partially due to variations in preparation methods remains unanswered. This indicates that while black tea has the potential to exert antioxidant effects, its efficacy may be influenced by various confounding factors. Therefore, further investigation into optimal consumption conditions is necessary. The anti-inflammatory potential of black tea is similarly complex. Although some research indicates significant reductions in inflammatory markers and alterations in immune cell profiles in certain populations [52, 57], other studies did not report notable effects [51, 53]. Despite this, the interplay between black tea polyphenols and gut microbiota offers a promising avenue for future research. Published evidence suggests that black tea's effects can be modulated by microbiota [54] and, conversely, affect microbiota composition [55]. In this regard, we speculate that the divergent results observed in the reviewed trials could be partially attributed to individual differences in gut microbiota. This variability can affect flavonoid metabolism [54], thereby influencing the efficacy of black tea's bioactive compounds. Additionally, the alterations in specific gut microbial species following tea consumption, along with an enhancement in humoral immunity [55], indicate potential novel mechanisms by which black tea may offer health benefits. These findings merit further investigation.

The primary strength of this review is its exclusive focus on black tea, supported by data obtained from clinical trials. However, there are several limitations to the existing evidence base. First, several of the trials examined are characterized by small sample sizes and short durations, and there is significant variability in study methodologies, participant populations, and outcome assessments. Furthermore, the ideal dosage and preparation methods for black tea to maximize its health benefits have yet to be determined. Future research should prioritize larger, well-controlled trials with standardized interventions and extended follow-up periods. Second, this work is a narrative review, which may be susceptible to selection bias in the studies included. While not without limitations [60], conducting a systematic review with a comprehensive search strategy could offer a more thorough evaluation of the existing evidence. Nonetheless, narrative reviews are valuable for offering interpretation and critique, with their primary contribution being the enhancement of understanding [60]. Another limitation of this review is its focus solely on published literature, excluding searches of clinical trial registries like ClinicalTrials.gov. This approach may have led to the omission of relevant ongoing or completed trials that have not yet been published. Additionally, the potential for publication bias cannot be dismissed, as studies with positive outcomes are more likely to be published than those with null or negative results. This could result in an overestimation of the beneficial effects of black tea intake.

## 5. Conclusions

The aggregate evidence from clinical trials indicates that regular consumption of black tea may confer substantial health benefits across various domains. The observed enhancements in endothelial function, cognitive performance, mood, and stress are particularly noteworthy and merit further exploration. Although additional research is necessary to fully elucidate the underlying mechanisms and to establish optimal dosing strategies, incorporating moderate amounts of black tea (approximately 100 mg/day, equivalent to less than one standard cup) into a healthy lifestyle seems to be a promising strategy for enhancing overall well-being and reducing the likelihood of developing chronic conditions. These findings underscore the importance of continued exploration into the potential therapeutic applications of beverage interventions, such as black tea, for improving long-term health outcomes.

## Figures and Tables

**Table 1:** Search criteria applied in the narrative review.

**Criterion**	**Specification**
Date of search	July 1, 2024
Academic datasets	PubMed/MEDLINE, PubMed Central, Scopus, Web of Science, and Google Scholar
Search string	“black tea” AND (“clinical trial” OR “randomized controlled trial”)
Publication date	From January 1, 1990, to July 1, 2024
Eligible studies	Clinical trials or randomized controlled trials examining health effects of consuming black tea in clinical populations
Ineligible studies	Study design different from clinical trial or randomized clinical trials, non-English full-text articles

**Table 2:** Clinical trials assessing black tea in relation to cardiovascular health.

**Domain**	**Study**	**Population**	**Intervention**	**Outcome**
Endothelial and vascular function	Duffy et al. [16]	66 patients with coronary artery disease	Acute (450 mL) and chronic (900 mL/day for 4 weeks) black tea consumption	Improved FMD, effects attributed to tea flavonoids rather than caffeine
	Hodgson et al. [17]	21 subjects with mildly elevated cholesterol	5 cups/day (250 mL each) for 4 weeks	Increased endothelium-dependent (+2.3%) and independent (+4.2%) vasodilation
	Grassi et al. [18]	19 healthy males	Dose-dependent intake of black tea (≥ 100 mg/day)	Improved FMD, reduced arterial stiffness, and decreased systolic (−2.6 mmHg) and diastolic (−2.2 mmHg) blood pressure
	Hodgson et al. [19]	111 subjects with systolic BP 115–150 mmHg	3 cups/day for 6 months	Reduced nighttime blood pressure variability, sustained vascular benefits over long-term consumption
	Schreuder et al. [20]	20 healthy subjects	3 cups/day for 1 week	Improved FMD but no protection against ischemia–reperfusion-induced endothelial dysfunction
	Grassi et al. [21]	19 hypertensive patients	Acute black tea consumption	Increased circulating angiogenic cells, improved FMD, and counteracted fat-induced endothelial dysfunction
	Greyling et al. [22]	20 healthy middle-aged subjects	Acute black tea (400 mg flavonoids) vs. placebo	No significant effect on endothelium-dependent vasodilation of forearm resistance arteries, high variability in responses noted
	Woodward et al. [23]	20 healthy middle-aged adults	Acute black tea (200 mL) vs. placebo	Enhanced cutaneous vascular responses to gradual local heating, possibly via nitric oxide–mediated mechanisms
	Ahmad et al. [24]	17 healthy volunteers	Black tea with/without milk vs. hot water	Black tea alone improved FMD; adding milk reduced FMD and increased blood pressure

Cholesterol and lipid profile	Davies et al. [25], Fujita and Yamagami [26], Bahorun et al. [27]	Various populations with hypercholesterolemia	Black tea or extract tablets over different durations	Mixed results on lipid profiles: Reductions in LDL cholesterol observed in some studies, while others found no significant changes depending on dosage, duration, and population characteristics

Platelet aggregation and hemostasis	Hodgson et al. [28], Hodgson et al. [29], Steptoe et al. [30]	Healthy adults	Black tea vs. placebo	Limited effects on platelet aggregation and activation markers; soluble P-selectin levels decreased in one study but no consistent antiplatelet effects across trials were observed

Abbreviations: FMD, flow-mediated dilation; LDL, low-density lipoprotein.

**Table 3:** Clinical trials assessing black tea in relation to metabolic health.

**Domain**	**Study**	**Population**	**Intervention**	**Outcome**
Glucose metabolism/diabetes mellitus	Bryans, Judd, and Ellis [40]	16 healthy adults	250-mL water, water + caffeine, or water + 1.0-g instant black tea	Black tea reduced postprandial glucose at 120 min and increased insulin levels at 90 and 150 min compared to controls
	Butacnum, Chongsuwat, and Bumrungpert [41]	24 subjects with normal and prediabetic glucose levels	Sucrose solution with low (110 mg) or high (220 mg) doses of black tea polymerized polyphenols (BTPP) vs. placebo	BTPP significantly reduced postprandial blood glucose area under the curve; no significant insulin changes were observed
	Mackenzie, Leary, and Brooks [42]	49 adults with Type 2 diabetes	0, 375, or 750 mg/day of green and black tea extract for 3 months	No significant changes in glycated hemoglobin between treatment arms; no hypoglycemic effect was observed

Body weight	Bahorun et al. [27]	87 subjects	Daily consumption of 3 cups of black tea for 12 weeks	No significant changes in body weight or BMI compared to controls
	Troup et al. [38]	57 individuals with borderline hypercholesterolemia	5 cups/day of black tea for 4 weeks	No significant effects on body weight or BMI compared to placebo
	Bøhn et al. [43]	111 healthy adults	3 cups/day of black tea vs. caffeine-matched placebo for 6 months	Black tea inhibited weight gain (−0.64 kg), reduced waist circumference (−1.88 cm), and decreased waist-to-hip ratio (−0.03) over 3 months; effects were not sustained at 6 months

Abbreviations: BMI, body mass index; BTPP, black tea polymerized polyphenols.

**Table 4:** Clinical trials assessing black tea in relation to cognitive function, mood, and stress levels.

**Study**	**Population**	**Intervention**	**Outcome**
Hindmarch et al. [45]	Healthy adults	Black tea vs. placebo (no caffeine)	Improved accuracy on attention tasks and increased self-rated alertness, mitigated diurnal performance decrements found with placebo
De Bruin et al. [46]	Healthy adults	Black tea vs. placebo	Enhanced accuracy on switching attention tasks and boosted self-reported alertness
Okello, Abadi, and Abadi [47]	Healthy volunteers	Green and black tea consumption, EEG assessment	Increased alpha, theta, and beta brain wave activities postconsumption; green tea showed a stronger increase in theta waves
Yoto et al. [48]	Healthy adults	Inhalation of black tea aroma during stress-inducing arithmetic tasks	Reduced salivary chromogranin-A (stress marker) levels, decreased tension and anxiety scores immediately after exposure to black tea aroma

**Table 5:** Clinical trials assessing mechanistic underpinnings of black tea's health effects.

**Mechanism**	**Study**	**Population**	**Intervention**	**Outcome**
Antioxidant effects	Langley-Evans [49]	Healthy adults	Black tea with/without milk	Black tea without milk increased plasma antioxidant potential by 65%–76% over 6 h; milk attenuated this effect
	Bahorun et al. [27]	Healthy adults	3 cups/day for 12 weeks	Plasma antioxidant capacity increased by 418%
	Neyestani et al. [50]	Type 2 diabetes patients	Black tea extract (150–600 mL/day for 4 weeks)	Increased serum total antioxidant capacity and glutathione levels, suppressed malondialdehyde levels at higher doses (300–600 mL/day)
	Widlansky et al. [51]	Coronary artery disease patients	Acute and chronic black tea consumption	Increased plasma catechin levels but no significant effect on systemic oxidative stress markers

Anti-inflammatory effects	Bahorun et al. [52]	Type 2 diabetes patients	3 cups/day for 12 weeks	Significant reduction in C-reactive protein levels (53.4% in men and 41.1% in women), increased regulatory T cells and reduced proinflammatory markers
	Widlansky et al. [51]	Coronary artery disease patients	Acute and chronic black tea consumption	No significant changes in C-reactive protein concentrations or inflammatory markers
	de Maat et al. [53]	Healthy smokers	Black tea vs. green tea vs. placebo for 4 weeks	No significant changes in inflammatory markers across interventions

Gut microbiota effects	van Duynhoven et al. [54]	Healthy men	Black tea polyphenol metabolites (crossover trial)	Rapid absorption of catechins within 2–4 h, microbial catabolites sustained circulation, indicating gut microbiota involvement in health benefits
	Tomioka et al. [55]	Healthy Japanese adults	3 cups/day for 12 weeks	Increased *Prevotella abundance*, decreased fecal acetic acid, and enhanced butyrate-producing bacteria; improved salivary sIgA in participants with low baseline levels

## Data Availability

Data sharing is not applicable to this article as no datasets were generated or analyzed during the current study.
